# Digital connection, real bonding: Brief online chats boost interpersonal closeness regardless of the conversational topic

**DOI:** 10.1016/j.heliyon.2025.e42526

**Published:** 2025-02-07

**Authors:** Chiara Fini, Vanessa Era, Giovanna Cuomo, Ilenia Falcinelli, Mattia A. Gervasi, Matteo Candidi, Claudia Mazzuca, Marco Tullio Liuzza, Bodo Winter, Anna M. Borghi

**Affiliations:** a Department of Dynamic and Clinical Psychology, and Health Studies, Sapienza University of Rome, Rome, Italy; b Department of Psychology, Sapienza University of Rome, Rome, Italy; c IRCCS, Fondazione Santa Lucia, 00185, Rome, Italy; d Department of Medical and Surgical Sciences, “Magna Graecia” University of Catanzaro, Catanzaro, Italy; e Department of English Language and Literature, University of Birmingham, Birmingham, United Kingdom; f Institute of Cognitive Sciences and Technologies, Italian National Research Council, Rome, Italy

**Keywords:** Chat, Interpersonal closeness, Conversation, Abstractness, Self-other representation

## Abstract

This study explores how the quality of brief dyadic written exchanges (lasting under 5 min) on a virtual platform and the nature of the conversational topic (abstract or concrete), influences physical, interpersonal, and psychological closeness between interlocutors. In the first experiment, participants engaged in written conversations on either an abstract or concrete topic under two conditions: (i) an interactive condition, where participants exchanged messages with another person, and (ii) a non-interactive condition, where participants wrote independently on the same topic, aware that another person was simultaneously doing the same. Results indicated that participants in the interactive condition reported feeling significantly closer to their interlocutor than those in the non-interactive condition. In addition, greater perceived pleasantness, intimacy, and the importance of the other person's contribution to the conversation were associated with increased feelings of closeness. However, inconclusive evidence was obtained regarding the interaction of the other person's contribution with the abstractness of the conversational topic during the written exchanges in fostering feelings of closeness. The second experiment focused only on the interactive condition, where we examined interpersonal dynamics across different subcategories of abstract (e.g., philosophical/spiritual, emotional, social, physical/spatio-temporal) and concrete topics (e.g., tools, animals, food). The results of the first experiment were replicated, reinforcing the idea that the quality of the virtual exchange—rather than the topic itself—plays a crucial role in fostering feelings of closeness between individuals.

## 1 Introduction

### 1.1 New communication styles in the age of virtual platform

As of April 2023, 5.8 billion people (64.6 % of the world population) are estimated to be Internet users [1]. The rapid diffusion of virtual networking has triggered significant social transformations [2]. Social networks such as Facebook, Instagram, Whatsapp, Telegram, and Twitter are new platforms for self-expression and allow people to be constantly available to communicate with others [3,4]. The technological intrusion into the interpersonal sphere has caused important psychological and sociological phenomena. More than others, new communication styles are a direct consequence of the burgeoning of virtual written conversations [3]. The digital space allows people to start a conversation about a topic and create personal relationships. What are the main differences between virtual written conversations and traditional face-to-face ones? Corporeal proximity is no longer needed for virtual written conversations to take place. When chatting by writing messages on a computer screen, the richness of body language cannot contribute to smooth conversational dynamics. All kinds of interpersonal nonverbal signals and paralinguistic aspects (i.e., body language, gaze direction, voice tone, proximity, facial expressions) typical of face-to-face interactions [[5], [6], [7], [8]], are absent [9]. Despite this limitation, research reveals that virtual written conversations are effective in maintaining social ties and establishing new relationships [[10], [11], [12], [13]].

In written virtual conversations, where users engage in real-time conversations by sending messages that appear on the receiver's computer screen, the lack of embodied social feedback is partially compensated with alternative strategies (e.g., repeating the letters of some words, using emoticons, contractions, and acronyms [[14], [15], [16]]). These strategies illustrate the adaptability of human communication, reinforcing the understanding that, regardless of the (un)mediated modalities through which they are realized, conversations are a form of joint activity [17,18]. During a dialogue, two interlocutors try to accomplish the common goal of building new knowledge based on reciprocal conversational contributions. Without collaboration, a conversation becomes fragmented, chaotic, and ambiguous, and the information exchanged will not be fully understood by the interlocutors, as they lack a shared interpretative background, which can lead both to agreements or disagreements. Even though both face-to-face conversations and virtual written conversations are the product of collaboration efforts, the adopted interacting styles are quite different [4]. Various theoretical views attempt to frame these differences.

The hyperpersonal perspective claims that the absence of nonverbal cues, editing capabilities, identity cues, and temporal characteristics may lead interlocutors to favor self-presentation and partner idealization, fostering exchanges that are more intimate than those of face-to-face counterparts [19]. Similarly, the Social Identity and Deindividuation Theory [20] sustains that in virtual written conversations, reciprocal impressions are formed based on the social categories attributed to interlocutors rather than interpersonal cues. In line with the Uncertainty Reduction theory [21], Tidwell and Walther (2002) [4] found that in virtual written conversations, interlocutors employ more direct interactive uncertainty reduction strategies, such as asking intimate questions and sharing personal information, as compared with face-to-face conversations. Technology-mediated self-disclosure is at least as frequent and meaningful as face-to-face self-disclosure [22] and can lead to increased intimacy [23]. In conclusion, although qualitatively different from face-to-face conversations, virtual written conversations seem to foster interpersonal communication.

### 1.2 Fostering feelings of closeness through brief online chats: the role of the quality and the content of the dialogue

How do people experience virtual written conversations? Is a brief exchange enough to create a sense of closeness between strangers? Despite the growing prevalence of online communication, to date, research on the interpersonal dynamics promoted by virtual written conversations remains scarce. Since a conversation is a form of joint action, it may be guided by the same rules that govern joint activities. A recent study revealed that participants preferred to maintain a shorter distance from an unfamiliar individual when they imagined acting jointly (e.g., cutting together the same branch of a tree) compared to when they imagined acting in parallel (e.g. cutting separate branches of a tree) [24]. Based on this evidence, we might expect a similar pattern of results when the joint action corresponds to virtual written conversations.

Lahnakoski et al. (2020) [25] found that the interpersonal distance between the interactors predicted the quality of the interaction, with increased distance associated with lower enjoyment. Building on this, we reasoned that variables such as perceived pleasantness, difficulty attributed to the dialogue, intimacy, commitment, and self and other's contributions to the conversation, might all affect the quality of the interaction and, consequently, the interpersonal distance among the interlocutors.

As the body is not the privileged channel through which contents are conveyed in virtual written conversations, the quality of the interaction might depend on the topic of the conversation and the pragmatic digital rules governing the exchange. How much Self and Other identities representations have in common might capture the psychological closeness triggered by virtual written conversations. This is often measured using the Inclusion of Other in Self — iOS—scale [26,27]. Some studies have already exploited the iOS scale to measure interpersonal closeness after a social interaction [28,29]. According to Aron and Aron (1997) [27], the integration of what the other shares takes the name of ‘self-expansion’ and corresponds to greater relationship satisfaction and commitment. Conversely, physical-interpersonal closeness is captured by measuring the comfortable interpersonal distance between two individuals. This is typically assessed by asking participants to adjust the distance between avatars representing themselves and their partner, which reflects their preferred physical proximity. As we claimed above, in virtual written conversations, social signals in interpersonal linguistic exchanges pass through the conversational content and its chronometric [15] and typographic cues [16]. Hence, we also investigate whether the semantics of the conversational topic serve a pragmatic role: depending on the topic discussed, the interlocutors might be involved in different interpersonal dynamics, resulting in a different degree of perceived closeness.

### 1.3 Recognizing the other's contribution (when conversing about abstract vs concrete concepts) might lead to a feeling of physical and psychological closeness

Semantic knowledge is stored through concepts. Concrete concepts (e.g., “cat”, “chair”, “glass”) directly evoke sensorimotor experiences because they all point to an object reference and are easily imaginable [30]. Abstract concepts (e.g., “justice”, “freedom”, “peace”) [[31], [32], [33], [34], [35], [36], [37], [38]], instead, typically lack an object reference, are ubiquitous [39], and are characterized by stronger socio-linguistic components [40]. We can grasp the meaning of abstract concepts only when the interlocutors share a sociocultural sense-making background that provides interpretative coordinates. Finally, they evoke more interoception and emotions [[41], [42], [43], [44]]. The Words as Social Tool theory (WAT [31,32,34,[45], [46], [47]]) proposes that abstract concepts, as compared with concrete ones, are acquired and processed more through social interactions (for theoretical views, see Refs. [[48], [49], [50], [51]]), tested by applying an interactive paradigm [52,53]. In addition, a rating study has demonstrated that people perceive it as easier to start a conversation with an abstract as compared with a concrete topic, supporting the idea that, since they are acquired through linguistic exchanges, abstract concepts may better afford linguistic interactions [49].

Importantly, the WAT theory proposes social metacognition —asking for other's help to complement the knowledge about which people feel uncertain— as a potential mechanism involved during both the acquisition and the processing of abstract concepts. If this is the case, we would expect intellectual negotiations aimed at reducing uncertainty and more interactive exchanges to occur during conversations about abstract concepts [54]. Linguistic exchanges during conversations about abstract topics, indeed, might be conceptualized as *coordinated goal-directed and bidirectional actions*, where the goal consists of enriching each other with reciprocal knowledge.

In light of this, we can expect that the interpersonal dynamics between the interlocutors might be modulated by the conversational topic: abstract versus concrete. By recognizing the other person's contribution, individuals may become more committed to coordinating with the interactors [51], fostering a sense of physical and psychological closeness.

## 2 Experiment 1

### 2.1 Hypotheses and paradigm

In the first experiment, we aimed to test whether: Hypothesis 1) short written conversations vs simultaneous non-interactive writing on virtual platform increase the feeling of physical and psychological closeness between the interlocutors; Hypothesis 2) the quality of these conversations (pleasantness, commitment, other-contribution and self-contribution to the dialogue, difficulty, intimacy) further modulates the feeling of physical and psychological closeness. Finally, we intended to test whether: Hypothesis 3) the other's contribution to the conversation interacts with the abstractness of the conversational topic, fostering the feeling of physical and psychological closeness.

To test these hypotheses, we developed a new interactive paradigm requiring conversations on online platforms. The experiment included an *interactive* condition, in which participants conversed in dyads, and a *non-interactive* condition, in which they wrote about concrete or abstract concepts by knowing that on the other side of the screen, there was a person doing the same and that, later, they would read what the other had written. In the *non-interactive* condition, thus, participants knew that they were writing contemporaneously to another person and on the same content but did not interact with each other. After every conversation/verbal production, the psychological and physical/interpersonal closeness between the actors were measured. To measure the psychological closeness, we used the Inclusion of the Other in the Self (iOS) Scale [26]. To measure the physical/interpersonal closeness, we used a Visual Analogue Scale (VAS), where the distance between two avatars embodying the interlocutors could be adjusted, and participants were asked to stop their own or the partners’ avatar when they felt at ease with the reached interpersonal distance [55]. Together with these measures, participants were asked to evaluate on a Visual Analogue Scale (VAS) the pleasantness, the intimacy, the perceived difficulty experienced during the verbal production, and the self-contribution, plus the other-contribution and the commitment in the experimental interactive condition (covariates). We expected an increased physical and psychological closeness in the interactive compared with the non-interactive condition; in addition, we predicted that all the measured covariates might intervene in modulating the effects further. Regarding the conversational topic, we hypothesized that initiating a conversation about abstract concepts, compared to concrete ones, would require greater contributions from the other person. This cooperative effort in conversation might lead to increased physical and interpersonal closeness between the interlocutors.

### 2.2 Materials and methods

#### 2.2.1 Participants

Sixty-eight participants were recruited for the study. Participants were recruited through word of mouth/snowballing by master students in psychology. We created subgroups of participants matched for age and gender using a convenience sampling method. Five participants were excluded from the Interactive task as outliers (see ‘data analysis’ section), so the final sample included sixty-three participants (25 males, 38 females, group average age = 25.84 ± 5.28 years; group average years of education = 15.71 ± 2.8). The sample size was selected through a prospective power analysis performed with the software More Power [56]. We inserted as expected effect size a large partial eta squared value (0.14) [57]. Our analysis indicates that a within-subject design with one two-level factor, a power of 0.90, and a partial eta squared of 0.14 requires a sample size of 68 participants. The experimental protocol was approved by the ethics committee of the Department of Dynamic, Clinical Psychology, and Health Studies, Sapienza University of Rome (Prot. n. 0001040 November 16, 2020), and was carried out in accordance with the ethical standards of the 1964 Declaration of Helsinki and later amendments. **Participants provided written informed consent to participate in the study. While unaware of the study's specific aims: the theoretical hypotheses, they were fully informed about each step of the experimental procedures they would undergo. Keeping the research rationale implicit helped prevent response bias caused by compliance with the experimental method.**

#### 2.2.2 Stimuli

The stimuli consisted of eight abstract (i.e., *liberty, logic, challenge, mystery, future, ideal, fate, enigma)* and eight concrete concepts (i.e., *alarm clock, crown, station, diamond, crystal, canoe, muscle and bookcase).* Abstract concepts were selected from the Italian database [58]; concrete concepts were instead extracted from Italian database [59]. Abstract and concrete concepts were matched for some linguistic parameters, such as Familiarity based on the rating scores included in databases [58,59] (Concrete: *M* = 559, *SD* = 101.10; Abstract: *M* = 518, *SD* = 61.71; *t*(14) = 0.97, *p* = .22). Valence rating scores were obtained by asking two different groups of 26 and 30 participants to evaluate on a 7-point Likert scale 35 abstract and 35 concrete concepts each in terms of Valence. Evaluations were provided through the compilation of two online surveys implemented on Google Moduli. The sample size for each survey was estimated in line with that of previous works (i.e., a minimum of 20 participants- [58,60]). Then, 8 abstract and 8 concrete concepts not differing in their valence (Concrete: *M* = 4.01, *SD* = 0.6; Abstract: *M* = 4.25, *SD* = 0.86; *t*(14) = −0.63, *p* = .5) were selected.

#### 2.2.3 Online chat task

Pairs of same-gender participants were asked to chat online for 5 min through the ICQ platform (https://icq.com/desktop/en). Participants performed two main tasks, i.e. one *interactive* and one *non-interactive*. In the *interactive* task, each participant was paired with another participant to chat— by writing on the computer screen— about two abstract concepts, and then with a different participant, to chat about two concrete concepts. The order of the chat topic (abstract/concrete concepts, Category Factor) was counterbalanced across pairs. Before starting the experiment, different experimenters took care of calling each participant, to guide him/her in understanding the task procedure. Participants were required to switch off their mobile phones and to close every window on their computer, but the ICQ platform. Moreover, participants were instructed to chat about a specific topic assigned by the experiment, taking the turn at least two times each, and writing at least two lines of text for each turn. The total amount of time given to each pair to chat about a specific topic was 5 min. To promote a rich dialogue, participants were told that the fluency of their chat would have to be evaluated. Participants were not allowed to use emoticons while writing, to ensure that they verbally described emotions and mental states, which are important abstract concepts [58]. Additionally, since we aimed to understand how literal language in dialogue affects interpersonal dynamics, we wanted to ensure that participants use written words during their conversational turns. Once on the ICQ platform, participants were introduced to each other and started to chat.

In the *non-interactive task*, participants were instructed to write alone on the ICQ platform about a specific topic assigned by the experimenter. As for the interactive task, participants had to write in the chat at least two different sentences of two lines each. The total amount of time given to each participant was 5 min. To promote a rich production, participants were told that the fluency of their writing would have been evaluated. Participants were not allowed to use emoticons while writing. Once on the ICQ platform, each participant was introduced to a fake partner (played by the experimenter) and was told that the chat would not allow the person on the other side to read what the other was writing. Indeed, while writing, participants received the feedback “*The other is writing …*” from the platform page but did not see any text displayed. Thus, participants were writing the text alone, however, they were induced to think that another person was doing the same on the other side and that, at the end of the experiment, they would be allowed to see what the other wrote. Each participant performed both the interactive and non-interactive task, separated by a 2/8 weeks interval and their order was counterbalanced across participants. In both the non-interactive and the interactive task, each participant chatted/wrote about two abstract and two concrete concepts with two different other participants. To increase stimuli variability, different groups of participants used different set of concepts.

After each chat, participants answered to.

i) An interpersonal distance task [55]. The task allows for measuring the physical interpersonal closeness among interlocutors. A silhouette representing the participant was selected based on each participant's gender. The silhouettes were pictured in a standing position on a marker at the left end of a line and facing the right end of the line. The virtual avatars were realized using MakeHuman Community 1.2.0 (http://www.makehumancommunity.org); the pictures of the avatars were taken using Unity v.2019.4.15f1 (https://unity.com). Participants were asked to indicate a measure of passive and active distance. In the passive distance measurement, participants were provided with the following instructions: “Imagine that you are the person on the left of the line and that you cannot turn nor move. Then, imagine that the person you just chatted with, depicted on the right side of the line, begins walking toward you. You should indicate how close you would allow this person to approach you while still being comfortable with that distance. To indicate where the other should stop, click on the horizontal line. Then, press ‘Next’ to proceed”. In the active distance measurement, participants were presented with the following instruction: “In another condition, you will be asked to indicate how close you would get to the other person by imagining that you are moving in the same horizontal line. Click to report where you would like to stop, while still being comfortable with that distance. Then press ‘Next’ to proceed”.
ii) The Inclusion of Other in Self (iOS) Scale [26], aimed at measuring psychological closeness among interlocutors. In the scale, participants are presented with a single item, where six configurations are displayed. In each configuration, two circles, representing the Self and the Other, are positioned at an increasing smaller distance, until they overlap. Participants are asked to indicate which configuration best represented their perceived relationship with the person they just chatted with.
iii) Different VAS scales, ranging from 0 to 100, regarding some aspects of the previous experience of chatting: the perceived Other-contribution, the Self contribution (only for the Interactive task), the degree of Intimacy, the Difficulty, the Commitment, and the Pleasantness of the chat. All the VAS scales were used to measure the quality of the virtual written conversation in the interactive condition, while the other four VAS (without self and other-contributions) scales measured the perception of the quality of written production in the non-interactive condition.

**Fig. 1:**
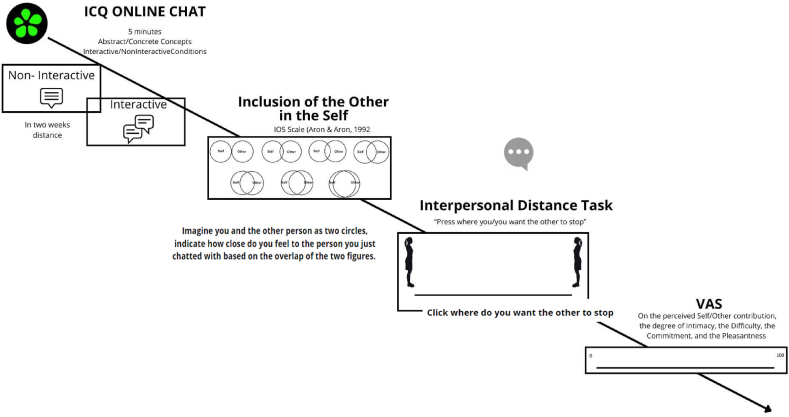
Timeline of the experimental procedure. Participants were asked to chat contemporaneously with another person (interactive condition - top left image), or to individually write (non-interactive condition - top right image) about an abstract or concrete topic. After each chat, participants were asked to perform the interpersonal distance task (bottom right image) and to choose the most representative configuration of the Inclusion of Other in Self (iOS) Scale (bottom left image) and some VAS scales regarding the perceived Other-contribution, the Self contribution (only for the Interactive task), the Intimacy, the Difficulty, the Commitment and the Pleasantness of the previous chat.

Fig. 1 shows a graphic representation of the experimental timeline.

### 2.3 Data availability

All data and analysis code is available under the following publicly accessible Open Science Framework repository: https://osf.io/m39br/

### 2.4 Data analysis

Data were analyzed through R (version 4.1.1 - R-Core Team, 2019) in RStudio (version 4.3.1). ‘Tidyverse’ (version 1.3.2 -^61^) R's package was used for data processing and visualization. For creating multi-plot arrays, we used ‘patchwork’ (version 1.1.1 -^62^) and ‘gridExtra’ (version 2.3 -^63^) R's packages. ‘Joy’ or ‘ridge’ graphs were plotted with ‘ggridges’ (version 0.5.3 -^64^) R's package. Finally, Bayesian generalized linear mixed effects models were fitted using ‘brms’ (version 2.16.2 -^65^) R's package.

Before analyzing the data, we first assessed whether the closeness ratings differed between the ‘active’ and ‘passive’ questions. A simple Spearman's rho correlation showed that the two were highly correlated (*rho* = 0.92), which made us combine the two types of questions into a singular ‘closeness’ variable by taking the average. A total of 8 participants (80 data points, 7.4 % of the total data) were excluded from all further analysis, based on the criterion that they were below or above 2 standard deviations from the sampled average in the physical/interpersonal distance. If these participants were kept in the sample we analyzed, our primary conclusions would not be affected.

Our main analysis focuses on two dependent variables: iOS and interpersonal closeness. For all models where the dependent variable is the Inclusion of Other in Self (iOS) Scale, which is ordinal with six scale points, we use a mixed ordinal logistic regression, specifically, cumulative link models [61] with logit link function. For all models where the dependent variable is “closeness”, which is a visual analogue scale bounded by [0, 1], we used mixed beta regression with mean/phi parametrization. The beta distribution is commonly used for dependent variables that are continuous but bounded to a finite range, such as proportions and percentages.

Our first analyses are targeted at assessing the iOS scale. In a first analysis, we regress all covariates (pleasantness, commitment, intimacy, difficulty, self-contribution, other-contribution) onto an ordinal logistic regression model with random intercepts for participants and items and by-participant varying slopes for all covariates. This analysis was repeated for the interpersonal closeness measure (mixed beta regression, random effects structure the same). These analyses also informed our choices about which covariates to include in the main model. We then assessed whether closeness and iOS differ as a function of condition (interactive versus non-interactive). For both of these variables, models contain the critical fixed effects “condition” and no other fixed effects. The random effects are subjects and items, with both random intercepts and random slopes for the condition effect. The main two models were (cumulative ordinal) iOS or interpersonal closeness (beta) regressed onto the fixed effects ‘concept category’ (abstract versus concrete), the continuous covariate ‘other-contribution’ (centered), as well as their interaction, ‘category ∗ other's contribution.’ We centered the continuous other-contribution covariate to more easily interpret the abstract versus concrete category effect (at the mean of the other-contribution covariate). We decided to leave the default treatment coding system for ‘category’ (0 = abstract, +1 = concrete) as we are not specifically interested in a main effect of iOS. Random effects included random intercepts for participants and items, as well as by-participant varying slopes for all fixed effects, and by-item varying slopes for category. As discussed in the results section, the fact that abstract concepts also showed a trend to lead to interactions that were perceived to be slightly more difficult made us decide to include this as an additional covariate, which is the final model we used.

We used default priors assigned by brms except for beta coefficients, for which we chose weakly informative priors (for further detail, see the Open Science Framework repository: https://osf.io/m39br/). With our variables and tasks being fundamentally novel, we had little prior research to inform our prior choices. We therefore followed Gelman et al.’s (2008) [62] recommendations for a sensible default for weakly informative priors in logistic regression, which uses the same link function as our models. We set Cauchy priors centered at zero with scale = 2.5 on all beta coefficients. These priors weakly bias coefficients towards zero, following the assumption that most effects tend to be small. This builds “mild skepticism” [63] into our model, making results more conservative. Conclusions are not altered from models with flat priors on coefficients that do not make any prior assumptions about effect size, except for the Bayes factor analysis, which is known to be highly sensitive to prior choices. With flat priors, the main interaction we report to have strong evidence for the null in Experiment 1 (Bayes Factor = 0.06) only has “anecdotal” (0.6) evidence against the null; in Experiment it changes from “strong” evidence against the null to “anecdotal”. However, these changes do not alter our theoretical conclusions, as we would still report a lack of strong evidence for an interaction in either direction. For all remaining parameters in the model, we used default priors chosen by brms, i.e., only the priors for the fixed coefficients have been manually changed. The results section reports all models with weakly informative priors on beta coefficients.

All models were fitted with Markov Chain Monte Carlo, with 4 chains 3000 iterations used for posterior sampling (3000 warm-up samples were discarded). All models converged adequately (all Rhat = 1.0, all ESS >1000). We performed posterior predictive simulations (100 draws) to assess whether our models could plausibly have generated the data, which was the case for almost all models, except for a few models showing minor deviations between the simulated and raw data.

### 2.5 Results

#### 2.5.1 iOS and interpersonal closeness in interactive versus non-interactive condition

**Fig. 2:**
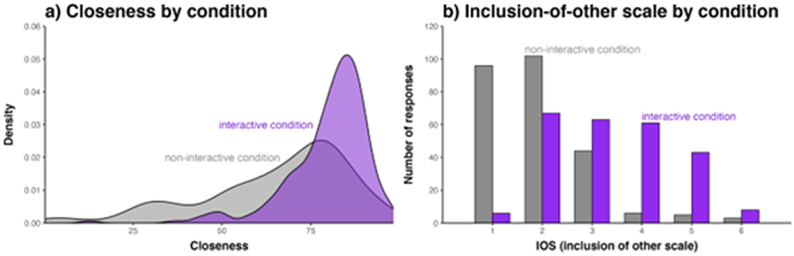
Panel a) Distribution of interpersonal closeness values (Visual Analog Scale). Panel b) iOS values as a function of condition.

In this section, we assessed whether physical-interpersonal and psychological closeness increase in the interactive vs non-interactive condition. Physical-interpersonal closeness increased in the interactive condition (M = 80.0) than in the non-interactive condition (M = 65.5), as shown in Fig. 2, Panel a). The mixed beta regression analysis indicates a positive effect of the interactive condition on interpersonal closeness (logit coefficient = +0.72, SE = 0.12) for which the 95 % credible interval is far away from zero: [0.47, 0.96]. Given the model and this data, the posterior probability of the interactive condition leading to a positive effect on interpersonal closeness is *p* = 1 (the entire posterior distribution lies above zero, with not a single sample indicating an effect of opposite sign).

Participants also gave higher iOS score ratings in the interactive condition (M = 3.37) than in the non-interactive condition (M = 1.95), as shown in Fig. 2, Panel b). The mixed ordinal regression analysis indicates a positive effect (+4.13, SE = 0.48) with a 95 % credible interval far away from zero: [3.21, 5.10]. Given the model and this data, the posterior probability of this coefficient being of the same sign was also *p* = 1. In addition, to explore the association between iOS and physical-interpersonal closeness we looked at the average closeness values for each of the six scale points of the iOS variable, which are 1 = 55.5, 2 = 75.4, 3 = 79.6, 4 = 81.0, 5 = 83.7, and 6 = 91.3. Clearly, higher iOS values correspond to higher closeness values. A mixed cumulative ordinal model where iOS values are regressed onto closeness with random effects for words and participants (including by-word and by-participant slopes for closeness) shows that closeness is a reliable predictor of which values participants choose on the iOS scale (logit estimate: +0.18, SE = 0.02, 95 % CrI: [0.13, 0.22]). The probability of this coefficient being of the same sign is, p = 1.0. The model including the random effects describes 65 % of the variance (R2); the Spearman correlation between both variables (without random effects) is rho = 0.45. We can, thus, conclude that iOS and closeness measure partially overlapping constructs, although they are not the same given a relatively substantial amount of variance that is left unaccounted for.

#### 2.5.2 How the quality of the dialogue impacts the feelings of physical and psychological closeness

Once shown that iOS and interpersonal closeness increased in the interactive vs the non-interactive condition, we explored whether the quality of the conversations impacted the physical-interpersonal and psychological closeness. We first explored how the covariates (pleasantness, commitment, intimacy, difficulty, self-contribution, other-contribution) behave with respect to each other, followed by how all covariates behave with respect to the iOS scale and interpersonal closeness. Table 1 shows Pearson's correlations between all covariates. The highest correlation was between ‘self-contribution’ and ‘other-contribution’ (*r* = 0.67), followed by ‘self-contribution’ and ‘commitment’ (*r* = 0.65), and ‘other-contribution’ and ‘pleasantness’ (*r* = 0.57). The ‘difficulty’ variable was either not correlated with any of the other variables, or negatively correlated, specifically, with ‘pleasantness’ (*r* = −0.29), and more weakly so, with ‘intimacy’ (*r* = −0.14).

**Table 1:** Pearson's correlations between all the covariates, i.e., Pleasantness, Commitment, Intimacy, Difficulty, Self-Contribution and Other-Contribution.

	Pleasantness	commitment	intimacy	difficulty	self-contribution	other-contribution
pleasantness		0.48	0.47	−0.29	0.43	0.57
commitment			0.45	0.00	0.65	0.47
intimacy				−0.14	0.47	0.41
difficulty					0.01	0.01
self-contribution						0.67
other-contribution						

**Fig. 3:**
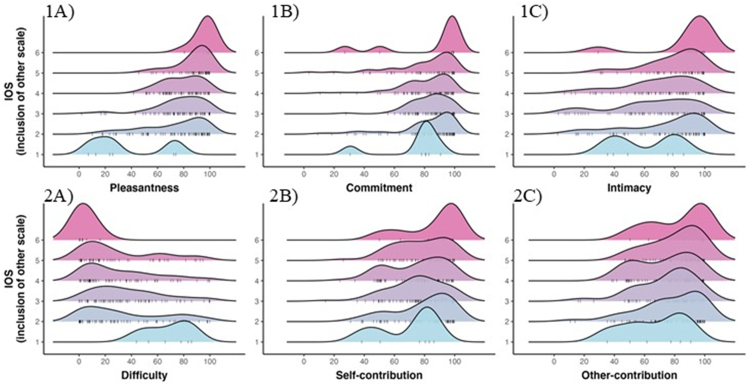
‘Ridge plots’ showing how all six covariates differ for different levels of the Inclusion of Other Scale (iOS).

Fig. 3 illustrates the distribution of each covariate for all levels of iOS. The figure shows that pleasantness (top left) differed greatly as a function of iOS. The highest iOS scale point had a pleasantness rating (M = 88.0) which was 79 % higher than the lowest point (M = 49.2). For commitment (top middle), the differences were much less pronounced, with the highest iOS scale point having a commitment rating of M = 87.2, compared to only M = 71.0 for the lowest value. Intimacy, on the other hand, differed more strongly as a function of iOS and also showed considerably more spread than the other two variables considered so far (top right). The highest iOS scale point had an average intimacy of M = 84.4; the lowest one of M = 59.3)

The highest iOS scale point was associated with the lowest difficulty level (M = 3.98), and the lowest iOS scale point was associated with the highest difficulty level (M = 68.4). A look at Fig. 3 (bottom left) reveals a noteworthy pattern, however: difficulty only differed starkly for the first and last iOS scale points, with less stark differences for the intermediate levels (2–5), and considerable spread. Either way, these results show that interactions judged to be difficult lead to lower feelings of other-inclusion. For the highest iOS scale point, self-contribution scores (M = 86.1) were somewhat higher than the lowest iOS scale point (M = 69.3), although this pattern was not very pronounced (Fig. 2, bottom middle). The other-contribution scores (Fig. 3, bottom right) were also higher for the highest iOS scale point (M = 83.0) than the lowest one (M = 66.6), with a similar spread to the self-contribution covariate. To assess uncertainty with respect to the patterns seen in Fig. 3, all covariates were regressed onto the iOS scale (with by-participant-varying random slopes for all covariates and word random intercepts). The ordinal logistic regression model revealed that pleasantness most strongly affected iOS (logit coefficient = +0.10, SE = 0.02), with a 95 % credible interval of this coefficient which was far away from zero: [0.07, 0.14]. The posterior probability of this effect being of the same sign was *p* = 1.0 (that is, every single posterior sample indicated an effect of the same sign). This means that given this model, data, and priors, there is little uncertainty about pleasantness being positively associated with iOS.

The only two other variables for which there was relatively little uncertainty included intimacy and difficulty. Intimacy was positively associated with iOS (+0.02, SE = 0.01, 95 % credible interval: [0.00, 0.04]), with a posterior probability of *p* = .97 of the coefficient having the same sign. Difficulty was negatively associated with iOS (−0.02, SE = 0.01, [−0.04, 0.00]), with a posterior probability of *p* = .96 of being of the same sign. Thus, higher pleasantness and intimacy, but lower difficulty, was associated with higher Inclusion of Other in Self (iOS) values.

**Fig. 4:**
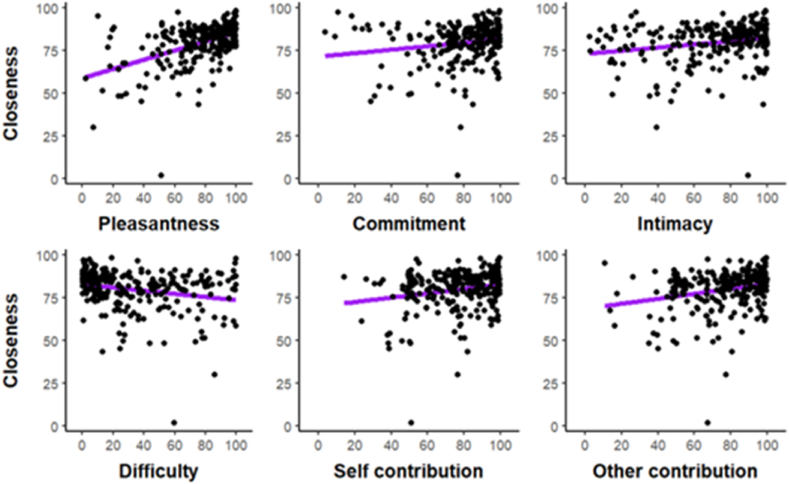
‘Regression plots’ showing how all six covariates differ for different levels of interpersonal closeness (VAS).

We repeated the same analysis for our second dependent variable, interpersonal closeness, which we regressed onto the same covariates (see Fig. 4). A similar set of covariates were associated with low uncertainty. Pleasantness was positively associated with interpersonal closeness (+0.01, SE < 0.01, [0.01, 0.02]), with a very high posterior probability of this coefficient being of the same sign, *p* = 1. Difficulty was negatively associated with interpersonal closeness (−0.01, SE < 0.01, [−0.01, 0]), with a very high posterior probability of being negative, *p* = .98. In contrast to the model, however, there was no indication of an effect of intimacy on interpersonal closeness, with the coefficient firmly centered on zero (+0.0, SE < 0.01, [0, 0]), and a posterior probability of the effect being of the same sign that was not informative, *p* = .52.

#### 2.5.3 The effect of the other's contribution (when conversing about abstract vs concrete concepts) on iOS and interpersonal distance

**Fig. 5:**
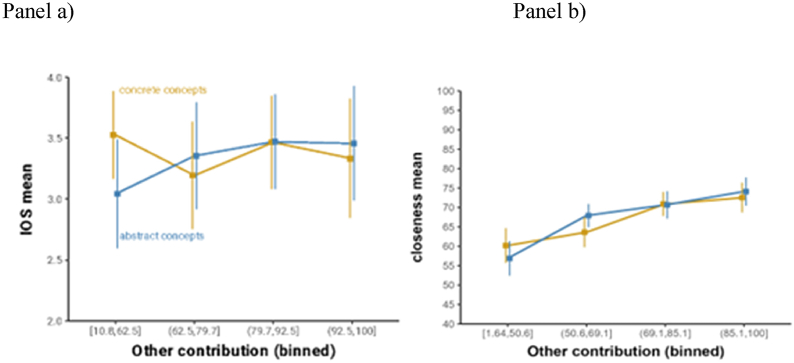
To visualize the descriptive pattern, the other contribution variable was split into four equal-sized bins; the error bars represent 95 % confidence intervals of the mean within each category.

We assessed whether there was an interaction between abstract concepts and the other-contribution scale with respect to the iOS score. Indeed, as mentioned above, we hypothesized that the other's contribution would be more relevant for attenuating uncertainties and negotiating meanings during conversations about abstract concepts. Fig. 5 displays this complex relationship by binning the other-contribution variable into four equal-sized groups with separate averages for concrete and abstract concepts. The standard errors are simple standard errors of the mean, which only serve heuristic value and do not correspond to our analysis [60]. To explore this relationship descriptively, we performed a Median Split on the other-contribution variable. For the high other-contribution set, abstract concepts had an average iOS of M = 3.46 compared to M = 3.40 for concrete. This pattern was reversed for the low other-contribution set, for which concrete concepts had a higher average iOS of M = 3.38 than abstract concepts M = 3.22.

The ordinal mixed model analysis revealed an interaction between category (abstract versus concrete) and other-contribution for the iOS dependent variable, with a positive coefficient (+0.06, SE = 0.03) indicating that for higher other-contribution values, specifically abstract concepts, led participants to respond with higher iOS values. This coefficient was close to but did not include, zero, with a 95 % credible interval: [0.01, 0.11]. The posterior probability of the coefficient being positive was high, *p* = .99. However, given the small effect size suggested by the descriptive averages and the pattern seen in Fig. 4, we decided to also compare the model with the interaction against the null model without the interaction using Bayes factors, which yielded BF = 0.06 ^1^, which corresponds to strong evidence for the null model (no interaction) against the full model (with interaction). R2 values of the model with the interaction were 1.3 % higher than the model without the interaction, and the 95 % credible intervals of these R2 values overlapped, which suggests that it is plausible that the interaction does not explain the variance. This model also includes difficulty as a covariate. There was no main effect of concept type (abstract *versus* concrete) on iOS values, with only a numerical trend for abstract concepts leading to lower iOS scores (logit coefficient = −0.20, SE = 0.5, 95 % CrI [−1.18, +0.80]). The posterior probability of this effect being of the same sign was 0.67.

[1] Bayes factors are scaled in the direction of the full model (with interaction) over the null model (without the interaction), which means that values > 1 indicate relatively more support for the full and <1 for the null model.

The other main dependent variable, physical-interpersonal closeness, also did not yield strong evidence for an interaction between other-contribution and concept category (logit estimate = 0.01, SE < 0.005, 95 % CrI [0.00, 0.02]), although a clear trend. The posterior probability of the interaction coefficient being positive was *p* = .94. In terms of main effects, other-contribution had a positive effect on interpersonal closeness (logit estimate = 0.01, SE < 0.005, 95 % CrI: [0, +0.02]), with a high posterior probability of being of the same sign, *p* = .98. Difficulty had a negative effect on interpersonal closeness (−0.01, *SE* < 0.005, 95 % CrI: [−0.1, 0]), with a high posterior probability of being of the same sign, *p* = 1. There was no main effect of concept category on closeness (logit estimate = −0.05, *SE* = 0.1, 95 % CrI: [−0.25, 0.15]), with an inconclusive posterior probability of being of the same sign, *p* = .69.

### 2.6 Discussion

The results of Experiment 1 are very clear. First, they show that participants felt psychologically and physically closer during the interactive than the non-interactive condition (Hypothesis 1). The results extend previous literature [24,27,64,65], and confirm the value of collaborative exchanges, like virtual written conversations, in modulating the perceived interpersonal space and the perceived overlapping between self-other representations [26]. When interacting with a new person through a social platform, individuals engage in an inferential process to predict the other's identity, personality, opinions, and values based on conversational content and emotional cues. This interpersonal inferential mechanism seems being sufficient to allow the establishment of a connection between humans [27], expressed by a sense of closeness both at a psychological and embodied-physical level. In addition, results show that linguistic interactions promote a sense of closeness already after 5 min of virtual written conversations. Interestingly, in keeping with our Hypothesis 2, the psychological closeness increased proportionally with the perception of how pleasant and intimate the exchange was perceived and decreased proportionally with how difficult the same was perceived. The perceived difficulty refers to the challenge of co-building a productive and coordinated new dialogical experience grounded on similar/different perspectives about the conversational topic. The more challenging a dialogue is perceived, the more likely people might experience an impasse, which can impact the flow of conversation and diminish the richness of the interaction. Interpersonal closeness [55], which is a more embodied and physical measure and indicates the imagined comfort zone from another person, was positively modulated by the pleasantness and the other's contribution to the dialogue and negatively modulated by the perceived difficulty, following a similar pattern as the iOS scale. Previous research demonstrated that the pleasantness of an interaction predicts the interpersonal closeness between two interactors [25] and that interpersonal closeness is modified by the quality of the social interaction and by positive/collaborative experiences [64,65]. Finally, regarding the abstract/concrete topic of the conversation, Experiment 1 did not provide evidence for the hypothesis that abstract concepts, requiring more of the other's contribution, lead to an increased closeness (Hypothesis 3). However, we only sampled eight concrete and eight abstract concepts, which, given the diversity of abstract concepts [58,66], could mean that we have dealt with a specific sample. We therefore decided to sample a larger variety of concepts in Experiment 2, as well as improve the precision of our inferences by also sampling a larger group of participants. Experiment 2 also improves on Experiment 1 by performing a subgroup analysis [58] for specific types of abstract concepts.

## 3 Experiment 2

### 3.1 Hypotheses and paradigm

Experiment 1 does not support the hypothesis that the more actors perceive interlocutors' contribution to a conversation about abstract topics, the more psychologically close they feel to the interlocutors. In the second experiment, we aimed to collect more robust evidence on the relation between the other's contribution and the abstractness of the conversational topics in fostering feelings of physical and psychological closeness. We also tested the impact of conversational quality (pleasantness, commitment, other-contribution, self-contribution to the dialogue, difficulty, intimacy) on modulating these feelings. In addition, we explored whether different subclusters of abstract (i.e., philosophical/spiritual, inner/emotional, self/sociality, and physical/spatio-temporal/quantitative) [58] and concrete concepts (i.e., tools, animals, food), selected as conversational topics, differently impact the feeling of physical and psychological closeness between the interlocutors. The experimental paradigm was identical to Experiment 1, except that the present one included only the interactive experimental condition. Indeed, after showing that conversing, as opposed to simply being aware of each other's presence on the social platform, promotes physical and psychological closeness, we were interested in exploring in more detail whether the conversational topic might modulate this closeness. In this regard, a recent study has shown that during simulations of conversational exchanges, in which participants responded to sentences involving different sub-kinds of concrete (i.e., tools, animals, food) and abstract concepts (i.e., philosophical/spiritual, inner/emotional, self/sociality, and physical/spatio-temporal/quantitative), the content of the verbal production changed [58]. Specifically, in a simulated conversation, abstract concepts generated higher uncertainty and more interactive exchanges than concrete ones; in a sentence plausibility task, participants considered uncertainty follow-ups (e.g., “What do you mean?” vs. “Well-done”) and follow-ups indicating curiosity (e.g., “tell me more” vs. “ok”) more plausible with sentences including abstract than concrete words [54]. In keeping with these results, our aim here was to better explore how prompting conversations with concepts of different natures might affect the sense of closeness between the interlocutors. As declared in the preregistration, we expected to find that the higher a participant rates the other-contribution to the conversation, the higher the interpersonal closeness between the interlocutors when conversing about abstract concepts but not about concrete ones. In addition, we expected to find that the other-contribution would impact the psychological/physical interpersonal closeness depending on the subkind of abstract concepts. Experiment 2 sampled a wider range of concepts from different subgroups of abstract concepts. Moreover, given indications of a weak effect in Experiment 1, we increased our sample size based on a power analysis. Experiment 2 hypotheses, methods, and analyses were formally pre-registered (https://osf.io/ynuew). As declared in the preregistration, here we aim to provide stronger evidence about the trend found in Experiment 1 —the more actors perceive interlocutors' contribution to a conversation about abstract topics, the more psychologically close to the interlocutors they feel— and to explore the modulation of iOS and interpersonal-physical distance by different sub-clusters of abstract concepts. We have to clarify that, although in the preregistration we declared that for data analysis it would have adopted a frequentist approach by applying Linear Mixed Model (LMM) with the maximal random structure, considering that many models were not converging, we decided to shift to a Bayesian approach.

### 3.2 Material and methods

#### 3.2.1 Participants

One-hundred and thirty-six participants were recruited for the study. Seven participants were excluded from the Interactive task as outliers (see ‘data analysis’ section), so that the final sample included one hundred and twenty-nine participants (58 males, 71 females, group average age = 25 years; group average years of education = 16). The sample size is based on a power analysis conducted with the software MorePower 6.0.4^56^ for a five level, one factor within participants design. The analysis indicates that the sample size is adequate for detecting a small to moderate effect size ηp2 = 0.035, with a power of 0.95 and an alpha level (two-sides) of 0.05. The experimental protocol was approved by the ethics committee of the Department of Dynamic, Clinical Psychology and Health Studies, Sapienza University of Rome (Prot. n. 0001040 November 16, 2020), and was carried out in accordance with the ethical standards of the 1964 Declaration of Helsinki and later amendments. Participants provided written informed consent to participate in the study. While unaware of the study's specific aims: the theoretical hypotheses, they were fully informed about each step of the experimental procedures they would undergo. Keeping the research rationale implicit helped prevent response bias caused by compliance with the experimental method.

#### 3.2.2 Stimuli

The stimuli consisted of sixteen abstract (*Beginning, Thrill, Competition, Sum, Enigma, Fame, Cause, Trend, Discovery, Oath, Habit, Attention, Trick, Tale, Silence, Pride*) and sixteen concrete concepts (*Fisherman, Mirror, Bookcase, Pumpkin, Tailor, Uniform, Oak, Tourist, Aeroplane, Cock, Table, Bottle, Notebook, Pot, Cactus, Salad*). Abstract concepts were selected from the Italian database [58], concrete concepts were instead extracted from two Italian databases [59,67]. These stimuli were selected through a previous validation survey in which 112 participants, divided in four groups, were asked to evaluate on a 7-point Likert scale, 149 abstract and 157 concrete concepts (divided in four different surveys) in terms of valence. Concrete concepts were divided a posteriori in two macro categories: “organic” and “inorganic” and we performed a Linear Mixed Model featuring as fixed factor Subclusters (concrete organic, concrete inorganic, Philosophical-Spiritual, Inner/Emotional, Self/Sociality, and Physical/Spatio-Temporal/quantitative), as dependent variable Valence ratings and as random intercepts Participants and Words. The model did not yield a significant effect of Subcluster, *F*(4, 24.53) = 0.6068, *p* = .662), showing that subclusters did not differ among each other.

#### 3.2.3 Online chat task

The experimental procedure was the same as Experiment 1, with the only difference that here there was only the interactive condition.

### 3.3 Data analysis

The statistical analysis for Experiment 2 largely mirrored that of Experiment 2. One noteworthy change is that we additionally performed a subgroup analysis on the abstract concepts only, looking at differences between the four subclusters of abstract concepts. All models include perceived difficulty as a covariate.

### 3.4 Results

#### 3.4.1 Recognizing the other's contribution (when conversing about abstract vs concepts) on iOS and interpersonal distance

**Fig. 6:**
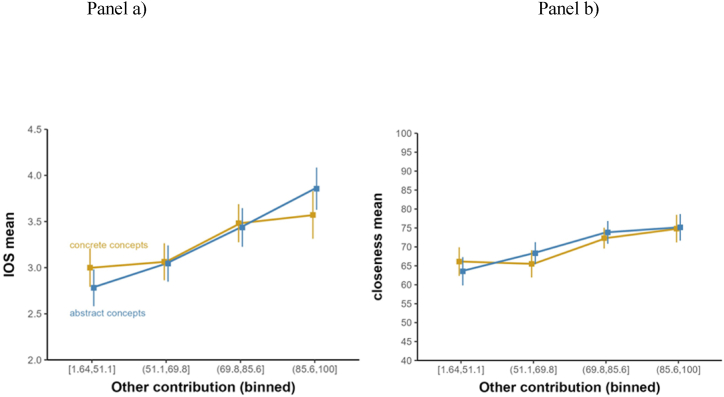
To visualize the descriptive pattern, the other-contribution variable was split into four equal-sized bins; the error bars represent 95 % confidence intervals of the mean within each category.

In this second experiment, we collected more solid evidence for the main hypothesis about the interaction between category and other-contribution on iOS. Our hypothesis was not confirmed. Fig. 6 provides a visualization of the absence of an interaction between other-contribution (x-axis) and concept categories (separate lines).

For the mixed logistic ordinal model, the coefficient of the interaction was ∼0.0, with a 95 % credible interval centered on zero: [−0.02, +0.03]. The Bayes factor comparing the model with the interaction against the model without the interaction indicated weak-to-negligible evidence in favor of the null model without the interaction, BF = 0.91. The two models were also equivalent in R2. Fig. 6 shows that in Experiment 2, there was a clear trend with higher other-contribution values leading to higher iOS values. This was reflected in the model in a positive coefficient of other-contribution (+0.06, SE = 0.01), with a 95 % credible interval far away from zero: [0.04, 0.08], the posterior probability of being positive *p* > 0 = 1. The difficulty covariate was also negatively associated with iOS (−0.03, SE < 0.005), with a 95 % credible interval that did not overlap with zero, [−0.04, −0.02], and a high posterior probability of being negative *p* < 0 = 1.0. As was the case in Experiment 1, there was no indication of a main effect of concept category (+0.02, SE = 0.21), with a 95 % credible interval firmly centered at zero: [−0.40, +0.43], *p* > 0 = 0.54). Similar results were obtained for the model with interpersonal closeness as a main dependent measure, as well as with interactions with concept category and self-contribution on either iOS or interpersonal closeness (all posterior probabilities of the interaction coefficient being of the same sign 0.20–0.80). Zooming in to the subgroups of abstract concepts, the pattern was similar, in that there was no indication of any interaction effects for other-contribution for any of the four subgroups of abstract concepts. All coefficients were close to zero (between −0.01 and + 0.01) and associated with large standard errors (SE = 0.02–0.03) and 95 % credible intervals firmly centered on zero. Posterior probabilities of coefficients of the interaction coefficients being of the same sign ranged from 0.56 to 0.7, indicating that there was no compelling indication for any coefficient reliably excluding zero. The same was the case for the mixed beta regression model with interpersonal closeness as a dependent variable, where posterior probabilities of the interaction coefficients being of the same sign ranged from 0.64 to 0.71, all within the range of probabilities that do not firmly exclude zero. The same was the case when interactions were entered with self-contribution rather than other-contribution, for iOS as a dependent measure (posterior probabilities of interaction coefficients being of the same sign = 0.55-0.79) and interpersonal closeness as dependent measure (posterior probabilities = 0.67-0.85).

#### 3.4.2 How the quality of the dialogue impacts the feelings of physical and psychological closeness

In this session, we examined whether all the covariates, as in Experiment 1, influenced the feelings of physical and psychological closeness. Table 2 shows Pearson's correlations between all covariates. The highest correlation was between ‘self-contribution’ and ‘other-contribution,’ as well as between ‘commitment’ and ‘self-contribution (both: *r* = 0.64). Following this, the next highest correlations were obtained between ‘pleasantness’ and ‘other-contribution’ (*r* = 0.59), ‘pleasantness’ and ‘commitment’ (*r* = 0.54), and ‘pleasantness’ and ‘intimacy’ (*r* = 0.53). As was the case with Experiment 1, the ‘difficulty’ variable was negatively correlated with ‘pleasantness’ (*r* = −0.37), as well as more weakly so with all the other variables.

**Table 2:** Pearson's correlations between all the covariates, i.e., Pleasantness, Commitment, Intimacy, Difficulty, Self-Contribution and Other-Contribution.

	pleasantness	commitment	intimacy	difficulty	self-contribution	other-contribution
pleasantness		0.54	0.53	−0.37	0.45	0.59
commitment			0.53	−0.18	0.64	0.54
intimacy				−0.23	0.44	0.43
difficulty					−0.21	−0.19
self-contribution						0.64
other-contribution						

**Fig. 7:**
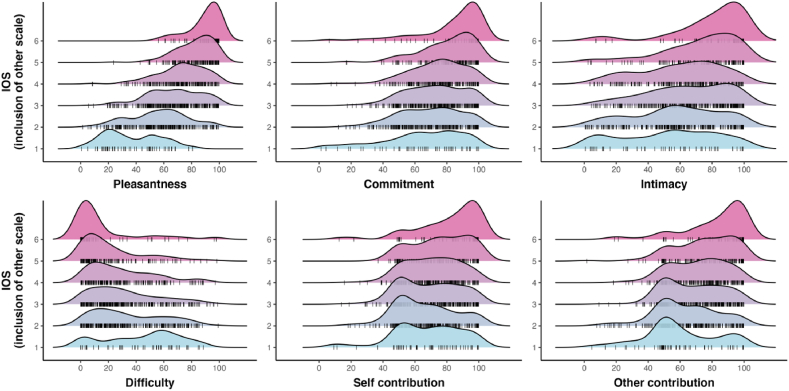
‘Ridge plots’ showing how all six covariates differ for different levels of the Inclusion of Other Scale (iOS).

**Fig. 8:**
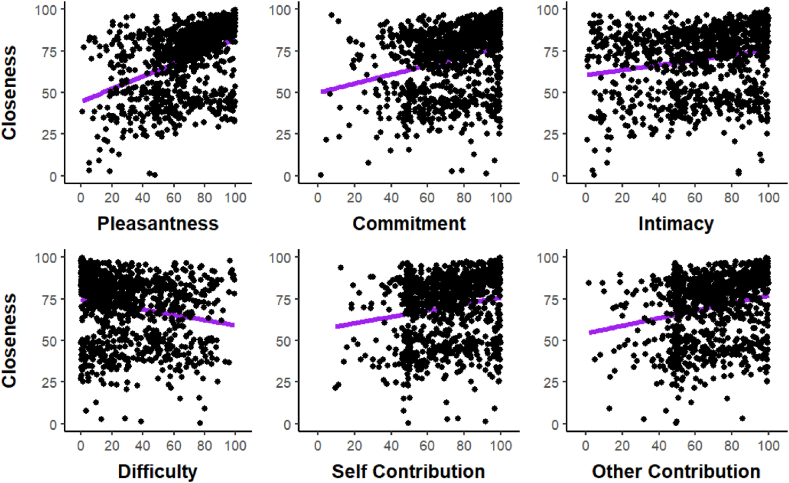
‘Regression plots’ showing how all six covariates differ for different levels of interpersonal closeness (VAS).

The overall pattern of how these covariates behaved with respect to iOS was similar to Experiment 1, as shown in Fig. 7, a mixed logistic ordinal model with iOS regressed onto all covariates showed a positive effect of pleasantness (logit coefficient = 0.09, SE = 0.01), with a 95 % credible interval far away from zero, [0.08, 0.11], that was associated with a high posterior probability of being positive *p* = 1.0. On top of this, intimacy was positively associated with iOS (+0.01, SE < 0.05, 95 % CrI: [0.00, 0.02], *p* > 0 = 0.98), and difficulty was negatively associated with iOS (−0.01, SE < 0.005, 95 % CrI: [−0.02, 0.00], *p* < 0 = 0.99). Similar results were obtained for interpersonal closeness in Fig. 8.

### 3.5 Discussion

Contrary to our expectations, the results of Experiment 2 suggest that psychological closeness (iOS) was not modulated by the degree of abstractness of the conversational topic, regardless of which subgroup of abstract concepts was considered. The same theoretical conclusions were furthermore obtained with the interpersonal closeness. The Words as Social Tool (WAT) theory hypothesizes that abstract concepts, compared with concrete ones, are acquired through socio-linguistic interactions. We, therefore, hypothesized that the need to reduce the uncertainty and to negotiate the meaning [49,54,[68], [69], [70]] might impact interpersonal dynamics. Our results, however, do not provide compelling evidence about the involvement of abstractness in interpersonal dynamics (e.g., psychological and interpersonal-physical closeness).

Several reasons may explain this lack of significant results. The first one is that we did not actually look into the conversations—this work will be the object of follow-up study—thus, we do not know whether the body of the conversation prompted by an abstract as compared with a concrete concept, holds also a more abstract level. Second, we did not apply any manipulations to control the abstractness level of conversation, for example stressing a common and “cultural” goal to achieve, which might ask for a deep understanding of conversational topics and thus prompt less free/spontaneous and more learning-centered interactions. Third, to allow the realization of a similar subtle interactive process, the virtual written conversations should have lasted more than 5 min; this may have helped to potentiate eventual discussions, negotiation, and opinion exchanges and would have been consistently expressed by the sense of closeness as a function of the conversational topic. Nevertheless, in Experiment 2, it emerges that, regardless of the topic's abstractness, the more participants considered as important the other's contribution to the conversation, the more they perceived themselves as psychologically and physically close to the other interlocutor. In other words, recognizing that the other plays a crucial role in linguistic exchanges leads to perceiving them as closer both at psychological and physical levels. Finally, the same results identified in Experiment 1 are replicated also in Experiment 2: the psychological closeness increased proportionally with the perception of how pleasant and intimate the exchange was perceived and decreased proportionally with how difficult it was perceived.

## 4 General discussion

In two experiments, we showed that virtual written conversations, lasting less than 5 min, are sufficient to increase the interpersonal-psychological and physical closeness between two interlocutors. We asked dyads to chat about a specific topic with another individual (interactive condition) or to write about the same topic through a social network (ICQ), knowing that on the other side of the screen, another person was doing the same and that s(he) would have read the text later (non-interactive condition). In the interactive condition, participants felt both psychologically and physically closer than in the non-interactive condition (Hypothesis 1). Our results shed light on the powerful effects of brief online written conversations in modulating the sense of closeness between two interlocutors. Psychological interpersonal closeness refers to the degree of perceived overlap between the self and other's mental representations (Inclusion of other in self scale [26]). The physical interpersonal closeness (measured through a Visual Analogue Scale–VAS– scale) refers to the representation of a hypothetical comfort spatial space between the self and the other - it is, therefore, an embodied measure, linked to the representation of the shared social space [71,72]. With the rise of non-traditional media and online social networking platforms such as Instagram, Facebook, and Twitter, there has been a significant shift in how people communicate and share information. Social media usage has increased dramatically in recent years [73], encompassing a wide range of activities: texting, emailing, information sharing, chatting, advertising, buying and selling, booking airlines and hotels, and learning. Such an innovative modality to stay in relation with others largely impacted our socio-emotional sphere and started a deep process of transformation of our mental interpersonal representations. A lot of research has already been conducted on how technology allows easy interaction and has the power to recalibrate the psychological distance— as it is intended by the Construal-level-theory [74]—among remote interactors [75,76]. It is known that mediated communication shapes perceptions of spatial distance [77] and promotes the so-called “death of distance” phenomena [78]: by bridging physical distance, virtual written conversations can decrease social distance [75]. Importantly, our results show that when two individuals chat in an “aspatial context”, like an online written chat, they represent themselves as closer compared to when they know about their reciprocal presence and write synchronously about the same topic, knowing that the other will read the text later. Our result points to different psychological mechanisms at work when virtually interacting. The term “Interactivity”, indeed, includes two scenarios: i) agents converse through virtual platforms —interactive condition— ii) an agent writes in the virtual platform (i.e., posting a link) —non-interactive condition— [79].

Conversing about a topic is a form of joint action [17,18] and requires a collaborative attitude to accomplish a shared goal: building together a new and rich discourse. If we conceptualize the conversation as a collaborative task, our results extend what has been already found during interpersonal sensorimotor interactions, to a virtual environment: imaging to collaborate on a common task increases interpersonal closeness between the interactors more than performing parallel actions [24]. Importantly, while *posting* something is typically a *self-centered approach*, *conversing* requires an *other-centered approach*. In this regard, Selfhout et al. (2009) [80] found that for adolescents who perceive low friendship quality, Internet use for communicative purposes (other-centered approach) predicted less depression, whereas Internet use for communicative (self-centered approach) purposes predicted more depression and more social anxiety.

Regarding the impact of the quality of conversations (pleasantness, commitment, other-contribution and self-contribution to the dialogue, difficulty, intimacy) on the feeling of physical and psychological closeness (Hypothesis 2) we found that the more the conversation was considered intimate in the Experiment 1, the more interlocutors felt psychologically closer, while, in Experiment 2 they felt both physically and psychologically closer. Intimacy [76] is related to physical proximity and the topic of conversation, and it is associated with self-disclosure. It is already known in the literature that virtual written conversations can even lead to greater intimacy than face-to-face communication [19]. For example, Bazarova (2012) [81] demonstrated that private disclosure prompted greater inferences of relational intimacy during on-line chats. There is evidence that, compared with face-to-face conversations, virtual written conversations might provide a unique context to compensate for the absence of nonverbal cues, as well as identity cues. According to the hyperpersonal perspective, these factors can contribute to the enactment of more intimate exchanges compared to their face-to-face counterparts (see the hyperpersonal perspective [19]).

Importantly, in both Experiments 1 and 2, we found that regardless of the conversational topic, both the physical-interpersonal and psychological closeness increased as a function of the pleasantness attributed to the conversation (Hypothesis 2). Our results are aligned with those of Lahnakoski et al. (2020) [25] who found that the interpersonal distance between the interactors predicted the quality of the interaction, with increased distance associated with lower enjoyment.

In the case of conversations, the pleasantness attributed to a verbal exchange depends on many factors, one of which is interlocutor's conversational sensitivity: the higher the sensitivity, the more high-level inferences when listening, the greater the ability to analyse individual conversational chunks, to keep them in memory, and to contribute personally to the dialogue. Conversational sensitivity is also positively related to self-monitoring, private self-consciousness, perceptiveness, self-esteem, assertiveness, empathy, and social skills [82].

Talking about abstract concepts might require more (linguistic) self-monitoring or inner speech during dialogue, as they are more complex than concrete ones and these processes might impact the interpersonal dynamics, such as the physical and psychological closeness between the interactors. In this regard, Borghi and Fernyhough (2023) [83] proposed three functions of inner speech for abstract concepts: the first one involves searching memory for the meaning of a concept—inner search/inner speech related to working memory. The second function involves monitoring/evaluating the correctness of the retrieved meaning—evaluative inner speech according to one's own knowledge. The third function involves preparing to ask another person for confirmation of the correctness of the retrieved information—dialogic inner speech. All these functions are strongly correlated with conceptual complexity. It might be that the selected abstract concepts were as familiar as the concrete ones, not requiring significant linguistic self-monitoring, searching meanings, or preparation to ask others in conversation. Being evaluated as equally difficult (see supplementary materials), and thus, requiring equal recruitment of cognitive resources, conversations might have been perceived as equally pleasant, regardless of whether the topic was abstract or concrete.

Conversing about abstract concepts might lead to more intellectually challenging conversations, thereby requiring more input from the other person to fuel and enrich the dialogue. However, we found that conversations about either abstract or concrete concepts were perceived as equally difficult in Experiment 1 (see supplementary materials). Importantly, Experiment 1 provided no evidence for the role of abstract concepts in leading to higher judgments of psychological closeness as a function of how much participants recognized the other's contribution to the conversation, and we replicated the same trend in Experiment 2 (Hypothesis 3).

The Words as Social tool—WAT— theory proposes that language and social interaction are more crucial to learn abstract concepts and that, due to their indeterminate meaning, abstract concepts promote social exchange and negotiation more than concrete concepts [32,[46], [47], [48],^70^]. Starting a conversation about an abstract concept would foster higher levels of negotiation and inferential process about the other's perspective and, consequently, an increased collaborative attitude in the dialogue, leading to interpersonal proximity. However, we found that the more participants thought that the other interlocutors contributed to the conversation, the more they felt psychologically and physically close to them, regardless of the conversational topic. Recognizing that the other person has actively participated in the dialogue, modulates interpersonal closeness and suggests the role of the spontaneous attribution of merit in reaching a shared goal: co-building knowledge can improve interpersonal closeness. The fact that the conversations were considered equally difficult, regardless of the topic, might explain why we did not find evidence for a relation between abstractness and the other's contribution to the conversation. Indeed, it might be that the more a conversation about abstract concepts is perceived as complex/difficult, the more the other's contribution to the dialogue might be perceived as relevant. In this regard, we cannot exclude that talking about more complex abstract meanings might lead to more difficult conversations, with a greater need for the other's contribution to attenuate uncertainties and doubts. In such challenging cases, the success of the conversation could potentially increase interpersonal closeness from the interlocutor. In conclusion, the present study shows that, in free conversations, starting from an abstract rather than a concrete concept does not lead to differences in perceived closeness with others. Why? The reasons can be many. First, it is possible that, being a form of joint action, conversation enhances closeness in any case, independent of the involved topic (unless in cases of conflict or different opinions). Another is that during a free conversation, which is not aimed at a specific goal (e.g., learning a new term, defining together a term, preparing actions based on the term knowledge, etc.), there is no need to reach a deep mutual understanding of the concept. Third, it is possible that even if the prompt for the conversation is a concrete vs. abstract concept, the flow of the conversation is directed elsewhere. Careful analyses of the conversation content will allow us to discover whether this is the case. Fourth, it is possible that analysing the content of the conversation, conversational repairs, etc. [84], differences between concrete and abstract concepts might emerge. Finally, it is possible that because the interaction is symmetric, the other is not crucial to enrich knowledge differently in the case of a concrete or an abstract concept. Further reasons for the absent result are possible. For example, we might be able to capture the needs of others with implicit but not explicit measures, such as the request to express a feeling of closeness. Further analyses and novel paradigms are necessary to test whether we need others more with abstract than concrete concepts in symmetric relationships.

### 4.1 Limitations of the study

In the current study, some limitations can be identified regarding the verification of the hypothesis about the modulation of the interpersonal dynamics between the interlocutors as a function of the abstractness of the conversational topic. First, the quality of the interaction between the interlocutors was spontaneous and in order to preserve that character, we did not control for the physiological content fluctuation typical of dialogical exchanges. By adopting such an ecological approach, we might have underestimated the need to exert manipulation on the conversational content. We did not control for the extension of the abstractness level of the initial topic to the entire body of the conversation. Second, the interactions, being so ecological, were less goal-directed and not performant; in other words, a deep understanding of the topic was not required in order to later re-explain the same to another person. For these reasons, the other's contribution to the conversation probably assumed a less utilitarian value: the goal to accomplish was not a “cultural/divulgation” one but the pure enjoyment of the conversation. Undoubtedly, the study suggests that conversations might represent diversified terrains of emotional and metacognitive experiences, allowing the speculation that some metacognitive experiences (i.e., other-contribution, self contribution, commitment) might interact with the abstractness of the topic and impact interpersonal dynamics in more performant-interactive scenarios as compared with spontaneous exchanges.

## 5 Conclusions

When conversing online, we are highly sensitive to the pleasantness, the difficulty, the intimacy, and the other's contribution to the dialogue. All these dimensions impact mental representations: people feel psychologically and physically closer when chatting compared to when they are just digitally connected with someone. Importantly, the subjective perception of the quality of the dialogue modulates the sense of closeness, while the topic of conversation is less relevant. Thus, we can conclude that virtual written conversations increase connection, but the *connection is not enough to feel authentically connected or closer* to someone; what matters is the dialogue and the subjective perception of its quality. Knowing the importance of talking online and conducting a qualitative dialogue, even if for a few minutes, can be particularly relevant to ameliorating the quality of life in contexts characterized by social isolation, mental illness, sickness, and for older people or hospitalized dwellers that cannot spend too much time in real social interactions due to their health conditions, but that can still benefit from this low-cost modality to feel closer and less alone.

## CRediT authorship contribution statement

**Chiara Fini:** Writing – review & editing, Writing – original draft, Supervision, Investigation, Methodology, Conceptualization. **Vanessa Era:** Writing – review & editing, Writing – original draft, Supervision, Methodology, Investigation, Conceptualization. **Giovanna Cuomo:** Visualization, Supervision, Data curation. **Ilenia Falcinelli:** Data curation. **Mattia A. Gervasi:** Data curation. **Matteo Candidi:** Methodology. **Claudia Mazzuca:** Writing – review & editing. **Marco Tullio Liuzza:** Data curation. **Bodo Winter:** Formal analysis. **Anna M. Borghi:** Writing – review & editing, Supervision, Funding acquisition.

## Inclusion and diversity statement

We support inclusive, diverse, equitable conduct of research.

## Fundings

This project was supported with funding from Next Generation EU, in the context of the National Recovery and Resilience Plan, Investment PE8 – Project Age-It: “Ageing Well in an Ageing Society”. This resource was co-financed by the Next Generation EU [DM 1557 October 11, 2022]. The views and opinions expressed are only those of the authors and do not necessarily reflect those of the European Union or the European Commission. Neither the European Union nor the European Commission can be held responsible for them.

BW was supported by the UKRI Future Leaders Fellowship MR/T040505/1.

VE was supported by Bando Giovani Ricercatori, Ministero italiano della Salute, GR-2021-12372923.

## Declaration of competing interest

The authors declare the following financial interests/personal relationships which may be considered as potential competing interests: Chiara Fini reports administrative support and article publishing charges were provided by University of Rome La Sapienza. Chiara Fini reports a relationship with University of Rome La Sapienza that includes: employment. Chiara Fini has patent licensed to Sapienza University of Rome. The authors declare to do not have any conflicts of interest.
