# Supplementary material for: Digital connection, real bonding: Brief online chats boost interpersonal closeness regardless of the conversational topic
Source: Heliyon. 2025 Feb 7;11(4):e42526. doi: 10.1016/j.heliyon.2025.e42526 (PMC11869026; doi:10.1016/j.heliyon.2025.e42526)
Supplement: Multimedia component 1 [file mmc1.docx]

SUPPLEMENTARY MATERIALS

We assessed the influence of abstract versus concrete concepts on perceived difficulty. Abstract concepts are perceived to lead to more difficult conversations (M = 36.6) than concrete concepts (M = 32.6) (see Figure 4). A mixed beta regression analysis on difficulty as the dependent measure indicates a positive effect of abstractness on perceived difficulty (logit coefficient = +0.07, SE = **0.20**), with a 95% credible interval that is clearly lopsided towards higher values [-0.34, **+0.46**], but that also clearly includes zero. The posterior probability of abstract concepts leading to higher difficulty ratings is p = 0.63. This means that although there is a numerical trend, given the model and the data, it is quite plausible that abstract concepts could also not increase perceived difficulty. Nonetheless, due to the numerical trend and the concern that people may only feel the need to depend on others because abstract concepts are more difficult, we decided to include difficulty as a covariate to see whether abstract concepts lead to higher (or perceived closeness) even when controlling for difficulty. It should be stated, however, that the results reported below hold regardless of whether difficulty is or is not included as a covariate.


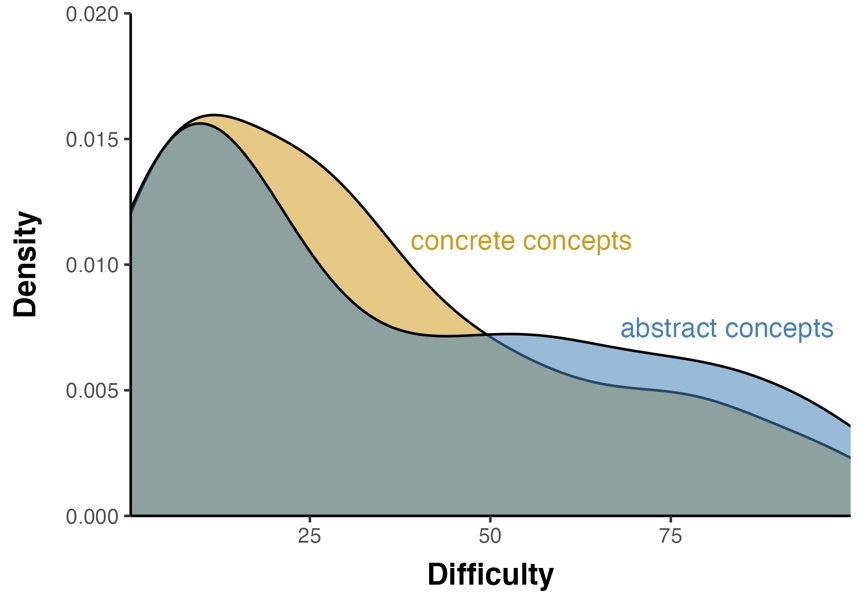


Figure 4. Distribution of difficulty ratings between concrete and abstract concepts.
